# Study protocol for a randomized, controlled, multicentre, pragmatic trial with Rehabkompassen®—a digital structured follow-up tool for facilitating patient-tailored rehabilitation in persons after stroke

**DOI:** 10.1186/s13063-023-07673-7

**Published:** 2023-10-06

**Authors:** Xiaolei Hu, Per Liv, Erik Lundström, Fredrik Norström, Olof Lindahl, Kristian Borg, Katharina S. Sunnerhagen

**Affiliations:** 1 Department of Community Medicine and Rehabilitation, Umeå University, Neuro-Head-Hals-Centrum, University Hospital of Umeå, Umeå, 901 87 Sweden; 2 Department of Public Health and Clinical Medicine, Umeå University, Umeå, Sweden; 3 Department of Medical Sciences, Neurology, Uppsala University, Akademiska Sjukhuset, Uppsala, Sweden; 4 Department of Epidemiology and Global Health, Umeå University, Umeå, Sweden; 5 Department of Radiation Sciences, Radiation Physics, Biomedical Engineering, Umeå University, Umeå, Sweden; 6 Division of Rehabilitation Medicine, Department of Clinical Sciences, Karolinska Institutet Danderyd Hospital, Stockholm, Sweden; 7 Department of Neuroscience and Physiology, Gothenburg University, Sahlgrenska University Hospital, Gothenburg, Sweden

**Keywords:** Stroke rehabilitation, Structured follow-up, Digital tool, ePROM, Effectiveness, Daily activity, Social participation, Cost-effectiveness, Health economy, Precision medicine

## Abstract

**Background:**

Stroke is a leading cause of disability among adults worldwide. A timely structured follow-up tool to identify patients’ rehabilitation needs and develop patient-tailored rehabilitation regimens to decrease disability is largely lacking in current stroke care. The overall purpose of this study is to evaluate the effectiveness of a novel digital follow-up tool, Rehabkompassen®, among persons discharged from acute care settings after a stroke.

**Methods:**

This multicentre, parallel, open-label, two-arm pragmatic randomized controlled trial with an allocation ratio of 1:1 will be conducted in Sweden. A total of 1106 adult stroke patients will have follow-up visits in usual care settings at 3 and 12 months after stroke onset. At the 3-month follow-up, participants will have a usual outpatient visit without (control group, *n* = 553) or with (intervention group, *n* = 553) the Rehabkompassen® tool. All participants will receive the intervention at the 12-month follow-up visit. Feedback from the end-users (patient and health care practitioners) will be collected after the visits. The primary outcomes will be the patients’ independence and social participation at the 12-month visits. Secondary outcomes will include end-users’ satisfaction, barriers and facilitators for adopting the instrument, other stroke impacts, health-related quality of life and the cost-effectiveness of the instrument, calculated by incremental cost per quality-adjusted life year (QALY).

**Discussion:**

The outcomes of this trial will inform clinical practice and health care policy on the role of the Rehabkompassen® digital follow-up tool in the post-acute continuum of care after stroke.

**Trial registration:**

ClinicalTrials.gov NCT04915027. Registered on 4 June 2021. ISRCTN registry ISRCTN63166587. Registered on 21 August 2023.

## Administrative information

**Table:** 

Title {1}	Study protocol for a randomized, controlled, multicentre, pragmatic trial with Rehabkompassen®– A digital structured follow-up tool for facilitating patient-tailored rehabilitation among persons after stroke
Trial registration {2a and 2b}	ClinicalTrials.gov Identifier: NCT04915027 ISRCTN registry with study registration number ISRCTN63166587
Protocol version {3}	2023 June, Version 5.0
Funding {4}	This study is funded by Swedish research consul (2022–00316 and 2022–00746); Västerbotten County Council and Umeå University (ALF Foundation, 2021–967513); Forte (2020–00136); the Heart–Lung Foundation (2,020,676); VINNOVA Medtech4Health (2019–01389); and the Swedish Stroke Foundation (Strokeförbundet)
Author details {5a}	**Xiaolei Hu** ^1^ Department of Community Medicine and Rehabilitation; Umeå University; Neuro-Head-Neck-Centrum, University Hospital of Umeå, Umeå **Per Liv** ^2^ Department of Public Health and Clinical Medicine, Umeå University, Umeå **Erik Lundström** ^3^ Department of Medical Sciences, Neurology, Uppsala University, Akademiska Sjukhuset, Uppsala **Fredrik Norström** ^4^ Department of Epidemiology and Global Health, Umeå University, Umeå **Olof Lindahl** ^5^ Department of Radiation Sciences, Radiation Physics, Biomedical Engineering; Umeå University, Umeå **Kristian Borg** ^6^ Karolinska Institutet, Department of Clinical Sciences, Danderyd Hospital, Division of Rehabilitation Medicine, Stockholm **Katharina S Sunnerhagen** ^7^ Department of Neuroscience and Physiology, Gothenburg University, Sahlgrenska University Hospital, Gothenburg, Sweden
Name and contact information for the trial sponsor {5b}	Hans Lindsten MD, PhD Head of the Neuro-Head-Neck-Centrum University Hospital of Umeå, 901 85 Umeå, Sweden Phone: + 46 90 785 0000 e-mail: hans.lindsten@regionvasterbotten.se
Role of sponsor {5c}	Neither the study sponsor nor funder have any role in the study design; collection, management, analysis, and interpretation of the data; writing of the report; or the decision to submit the report for publication

## Introduction

### Background and rationale {6a}

In Sweden, there are 100,000 stroke survivors and 23,000 new cases each year [1]. Persons with stroke often suffer heterogeneous functional impairments and limitations in various daily and social activities, followed by decreased health-related quality of life long after stroke onset [2, 3]. Unfortunately, no single rehabilitation regimen functions as a panacea for the diverse disabilities seen after stroke. In recent guidelines, the Swedish National Board of Health and Welfare has recommended that structured follow-up be provided to all patients with stroke [1] to identify their individual rehabilitation needs and develop patient-tailored rehabilitation regimens. This may enhance patient-centred care, which may thereby reduce health care costs without compromising quality or outcomes. However, such follow-up does not exist in current stroke care [4, 5]; instead, the post-acute continuum of care for stroke rehabilitation is considered inadequate and fragmented [4]. Moreover, the lack of knowledge of the cost-effectiveness of rehabilitation interventions has led to a prioritization of acute stroke care rather than rehabilitation interventions.

To understand patients and their heterogeneous disabilities, patient-reported outcome measures (PROMs) are widely used to promote patient-centred and patient-tailored care [6, 7]. Electronic PROMs (ePROMs) have further facilitated many of these positive effects in several other chronic diseases [8, 9]. An effective, user-friendly, digital and cost-effective PROM tool is urgently needed to facilitate patient-tailored rehabilitation regimens, strengthen patient-centred care and stitch together the currently fragmented post-acute continuum of care after stroke.

**Fig. 1:**
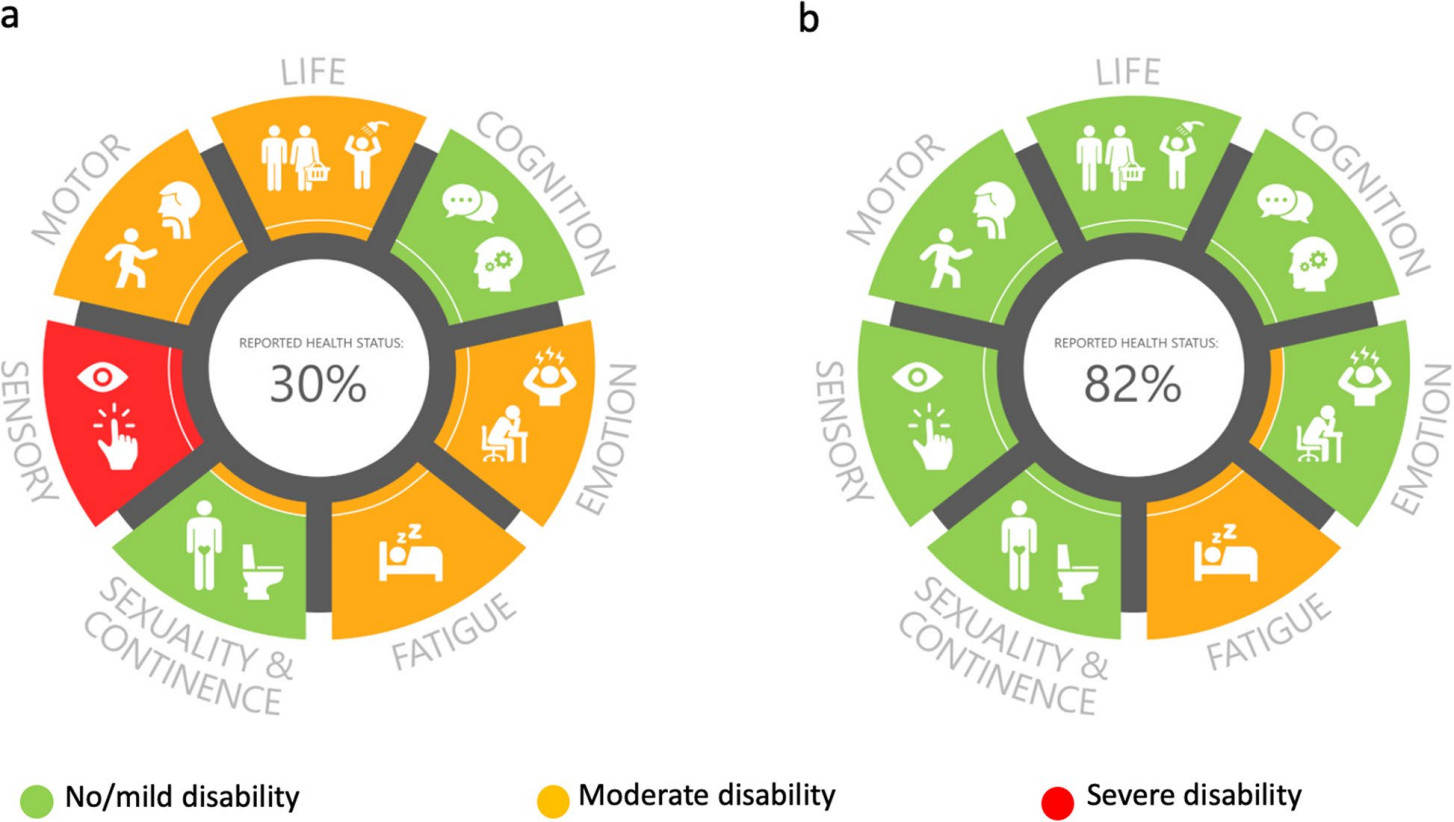
Rehabkompassen® identified more (**a**) or less (**b**) unmet rehabilitation needs at the 12-month follow-up after stroke onset for two different people. The figure published in our pilot study [11] is reused under a Creative Commons Attribution license

To meet these challenges, we created and developed a digital graphic follow-up tool, Rehabkompassen**®**, to embrace the heterogeneity of rehabilitation needs among stroke patients in real time (Fig. 1) [10, 11]; this digital tool is based on the paper-based version [12]. The Rehabkompassen® questionnaires consist of 6 well-validated and reliable PROMs, i.e. the simplified modified Rankin Scale questionnaire (smRSq); Stroke Impact Scale 3.0 (SIS) with additional questions related to sensory disturbances, sleep disturbances and natural topics; Hospital Anxiety and Depression Scale (HAD); Fatigue Assessment Scale (FAS); Eating Assessment Tool (EAT-10); and EuroQoL 5-dimension 5-level (EQ-5D-5L) [10, 12].

**Fig. 2:**
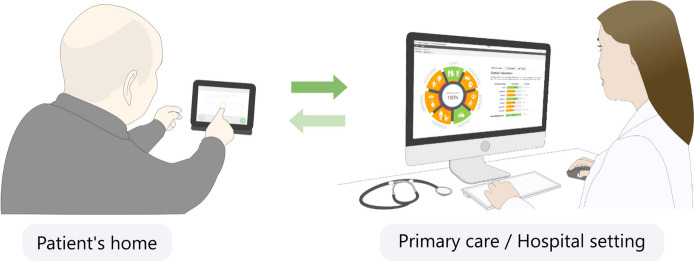
To use Rehabkompassen® in the health care system, a person with stroke completes the digital questionnaires, often at home, prior to a patient visit. The medical professionals can then assess the patient’s Rehab-Compass Graph before, during and after the visit. The figure published in our previous study [10] is reused under a Creative Commons Attribution license

The novelty of this instrument lies in its ability to provide an easy-to-understand and comprehensive picture of a stroke patient’s multidimensional problems/rehabilitation needs in a time-efficient way. Patients respond to Rehabkompassen**®** ePROM questionnaires at home 1 week before their clinical visit via the 1177.se website, which is *a Swedish* government-issued digital platform for Swedish citizens’ health care. The health care practitioners can thereafter directly visualize patients’ possible rehabilitation needs graphically on their computer (Fig. 2) even before the visit [10, 11].

By developing the novel Rehabkompassen**®** tool, we have combined patient-reported information and digital health technologies to facilitate the development of patient-tailored rehabilitation regimens and improve clinical outcomes in individuals after stroke. The overall purpose of this study is to evaluate the effectiveness of Rehabkompassen®, a novel digital follow-up tool, in persons discharged from acute care settings after stroke by comparing usual care with and without the Rehabkompassen**®**.

## Objectives {7}

### Primary research question

Does the incorporation of the digital Rehabkompassen® tool in usual care within 3 months after stroke onset result in improved daily and social activities for patients at the 12-month follow-up after stroke onset compared to those receiving usual care with the Post-Stroke Checklist (PSC)?

### Secondary research questions

The aim is to investigate the following by comparing usual care with the PSC ® (control group) to usual care with the usage of the digital Rehabkompassen**®** tool:

1. The improvement of technical and methodological aspects of Rehabkompassen® based on feedback from end-users, including both patients and health care practitioners
2. The provision of information that affects and/or facilitates the implementation of the Rehabkompassen® tool
3. The facilitation of triage, clinical assessments, decision-making, the development of a rehabilitation plan, referrals and outcome evaluations for health care professionals
4. The influence on secondary outcome measures, including fatigue, depression, anxiety and other stroke impacts, at the 12-month poststroke follow-up
5. The impact on patients’ health-related quality of life at the 12-month follow-up after stroke onset
6. The demonstration of the cost-effectiveness of the tool

## Trial design {8}

**Fig. 3:**
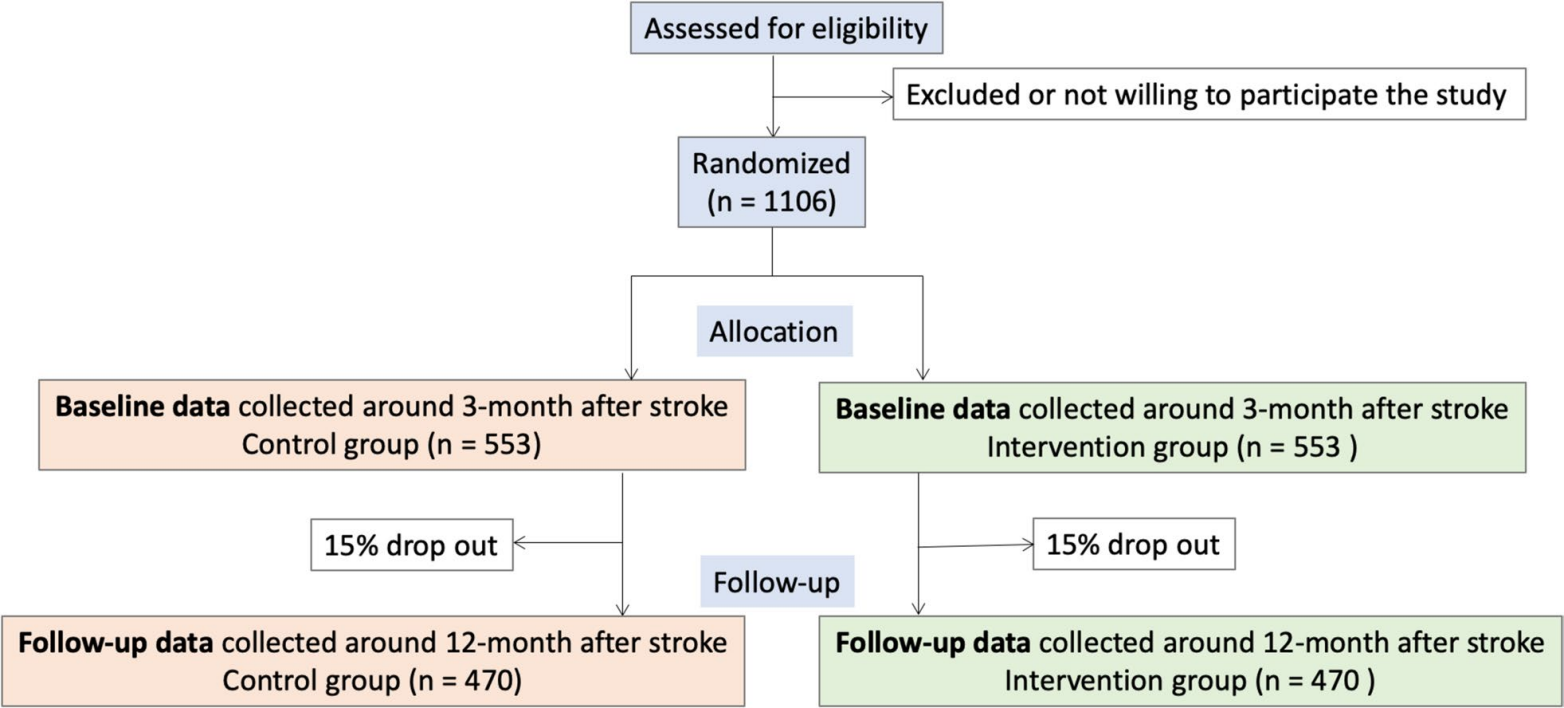
CONSORT flow chart

The Rehabkompassen study is a Swedish, parallel, open-label, two-arm prospective multicentre pragmatic RCT with an allocation ratio of 1:1 to investigate whether the usage of the novel digital Rehabkompassen**®** tool together with usual care is superior to usual care with the standard PSC as an active control (Fig. 3).

This trial protocol is reported in line with the Standard Protocol Items: Recommendations for Interventional Trials (SPIRIT) checklist [13] and the CONSORT statement for reporting pragmatic trials [14].

## Methods: participants, interventions and outcomes

### Study setting {9}

Participants will be recruited from approximately 15 centres across Sweden, including stroke units, early support discharge units and stroke rehabilitation units that provide care for persons within 3 months after stroke onset. Together with usual care, the control or intervention regimens will be flexibly delivered in in- or outpatient settings via either face-to-face or synchronous video visits in university, regional or local hospitals.

### Eligibility criteria {10}

#### Patient-participant eligibility

We aim to enrol as many stroke survivors as possible in usual care with little selection using the following criteria:

Inclusion criteria

1. Adults aged 18 years or older.
2. Time since stroke onset: individuals must be within the first 4 months after stroke, starting from day 1 after the occurrence of the stroke.
3. Patients discharged from acute care settings

Exclusion criteria

1. Unable to answer the evaluation questions
2. Unable to see the Rehabkompassen**®** graph
3. Not using BankID, an e-identification tool commonly used in Sweden

#### Clinical eligibility

Inpatient and outpatient clinics must meet the following eligibility criteria to participate in the study:

1. Have assignments for stroke patient follow-up and rehabilitation.
2. Able to provide in- and outpatient visits within 3 months and at 12 months poststroke.
3. Have a team with at least one physician and one nurse/other health care professional.
4. Have a referral system including both primary care and municipal care.

The reason for these requirements for participating clinics is to not only guarantee patient and staff resources but also to be able to study the cooperation among different care providers in the post-acute continuum of care after stroke.

#### Staff eligibility

Together with the Chief Investigator and the trial management group (TMG; see details in the “Composition of the coordinating centre and trial steering committee {5d}” section), the Principal Investigator (PI) at each site will ensure that all clinical staff, such as physicians, nurses, physiotherapists, occupational therapists and psychologists, who take part in the study are authorized, trained and competent according to the ethically approved protocol, principle of Good Clinical Practice (GCP) [15] and Declaration of Helsinki (1996) [16].

### Who will obtain informed consent? {26a}

All patients with stroke in the participating clinics during the study period will receive an invitation to participate in the study from the research personnel within 3 months after stroke onset. Written informed consent will be obtained by the research personnel prior to the participant undergoing study procedures. Research staff at the local clinic will contact patients who give their consent via telephone to provide oral information about the study, answer questions and determine whether the patients meet the selection criteria. The research staff will ensure that patients have sufficient time to consider their participation. All patients who meet the selection criteria and provide written consent will thereafter be randomized into the control or intervention group.

Study personnel will also provide their written informed consent prior to answering the questionnaires. Thus, written, informed consent to participate will be obtained from all participants.

### Additional consent provisions for the collection and use of participant data and biological specimens {26b}

No biological specimens will be collected in the study.

### Interventions

#### Explanation for the choice of comparators {6b}

A parallel, active control including the PSC with usual in- or outpatient visits is planned in the control group in the study. The PSC is a simple valid instrument for providing a structured follow-up for persons after stroke [17], which is recommended by Swedish national stroke guidelines [1]. The PSC [17] consists of eleven questions concerning common and treatable poststroke problems affecting the quality of life and has been used in the Swedish stroke population [18].

#### Intervention description {11a}

The intervention will consist of the usage of Rehabkompassen**®** and usual in- or outpatient visits in which a disease history will be taken, an examination will be performed and a rehabilitation treatment plan will be developed within 3 months after stroke onset*.* To implement the intervention, the Rehabkompassen program must be installed on the computers of the participating clinic. A 30–60-min introduction, demonstration and testing of the instrument will be provided to all personnel who are involved in the trial.

Participants in the intervention group will receive the Rehabkompassen**®** questionnaires in their inbox at 1177.se, *a Swedish*-government-issued digital platform for Swedish citizens’ health care. The patient-participants will complete the Rehabkompassen® questionnaires [12] by clicking on the links in their e-mail inbox at 1177.se. The questionnaires must be answered no later than 1 week before the 3-month visit. After completion of the questionnaire, the results will be exported by a research nurse to a secure server at the clinic and thereafter automatically transformed into a digital Rehab-Compass graph viewable to the physician and other medical staff at the clinic via the Rehabkompassen® tool on the computer (Fig. 2).

Similar to the pilot study [11], the tool can be used *before* the visit as a screening tool for initial triage depending on the colour coding of the patient’s personal Rehabkompassen® graph. During follow-up visits, health care practitioners will show the patients’ personal Rehabkompassen® graphs (Fig. 1) on the computer and use them as an illustration and communication platform to discuss the patients’ health status and rehabilitation needs. The personal Rehabkompassen® graph can also be used as a support tool in team meetings or for patient referrals *after* the visit.

All participants will use Rehabkompassen as a structured follow-up tool at the 12-month visit. In the intervention group, the patients’ personal Rehabkompassen® graphs at the 3- and 12-month visits allow assessment for evaluating the eventual effects of rehabilitation regimens or illustrating the changes in rehabilitation needs over time (Fig. 1) [10].

##### Postvisit assessments

After the 3- and 12-month follow-up visits, all patient-participants in both the intervention and control groups will answer a satisfaction questionnaire through 1177.se. The questionnaire will concern their overall experiences during the follow-up visit. Based on their needs, patients will receive various rehabilitation regimens according to the clinical routine after the visit, which will also be recorded by the health care practitioners via Research Electronic Data Capture (REDCap) [19] as clinical efficacy targets to monitor intervention fidelity in all participating clinics.

The health care personnel involved in the study will answer a questionnaire concerning their satisfaction and the specific perceived usability of the instrument in clinical practice via REDCap three times during the study period, i.e. at the beginning, middle and end of the study.

#### Criteria for discontinuing or modifying allocated interventions {11b}

There are no provisions for changing the trial arm allocation. However, participants may withdraw from the study without the need to provide a reason and their future care will not be affected. Any data collected up to the point of withdrawal will be included in the analyses.

#### Strategies to improve adherence to interventions {11c}

To optimize adherence to the study, we will provide various necessary help during the study. We are aware of some potential barriers for stroke patients, especially elderly individuals, to use the digital tool and answer many questions. Therefore, research staff will be available to offer technical support via telephone or in person when needed in addition to providing initial detailed instructions on how to answer the questionnaires. Since we consider that these questionnaires might be excessively burdensome for participants, the research staff member/nurse will encourage participants to take a break if they feel tired while completing them or to complete the ePROMs on different days. More advanced technical support during the study will be provided by technical staff on the research team. In addition, the 12-month visit is free of charge, which may improve adherence to the intervention.

The Chief Investigator and two research nurses will always be reachable during working hours for trouble-shooting and problem-solving during the study. Two RCT process controllers will regularly check all documentation of the participating clinics to ensure consistency among clinics. Annual trial meetings will be organized for all unit-allied health professionals for exchanging experiences and spreading knowledge. Monthly newsletters and ongoing e-mails will also be used for updates regarding the study progress and to facilitate communication during the study.

##### Strategies to improve the fidelity of the study

To minimize the differences among clinics, all unit-allied health professionals in the participating clinics will complete a training program on the usage of Rehabkompassen® in a setup phase before participant recruitment. To successfully implement the tool in broad routine clinical practice in the pragmatic RCT, a baseline survey concerning usual follow-up at each clinic will be completed before the start of the study, and any adjustment according to a usual care standard will be documented. Unit staff, including the multidisciplinary team, will participate in an orientation and information session about the study prior to the start of the study. Episodes of staff education and training will be provided for new staff on the unit. To implement and deliver the control and intervention regimens continuously, the participating clinics will receive annual economic compensation based on the number of participants who complete the study protocol.

#### Relevant concomitant care permitted or prohibited during the trial {11d}

No restrictions will be imposed on usual care during the study period. All rehabilitation treatments, including concomitant care and the intervention, will be documented at the 12-month follow-up.

#### Provisions for posttrial care {30}

No formal postcare provision is planned in the study. However, the rehabilitation needs identified at the 12-month follow-up will be treated according to the clinical routine in the respective participating clinics.

### Outcomes {12}

Participants’ demographic data (e.g. age, sex, comorbidities, stroke characteristics and severity) will be obtained via REDCap or from the Swedish Stroke Register (Riksstroke) at the first visit (T1 in Table 1). All data and outcomes collected by ePROMs will be in Swedish, which is the official language in Sweden.

**Table 1:**
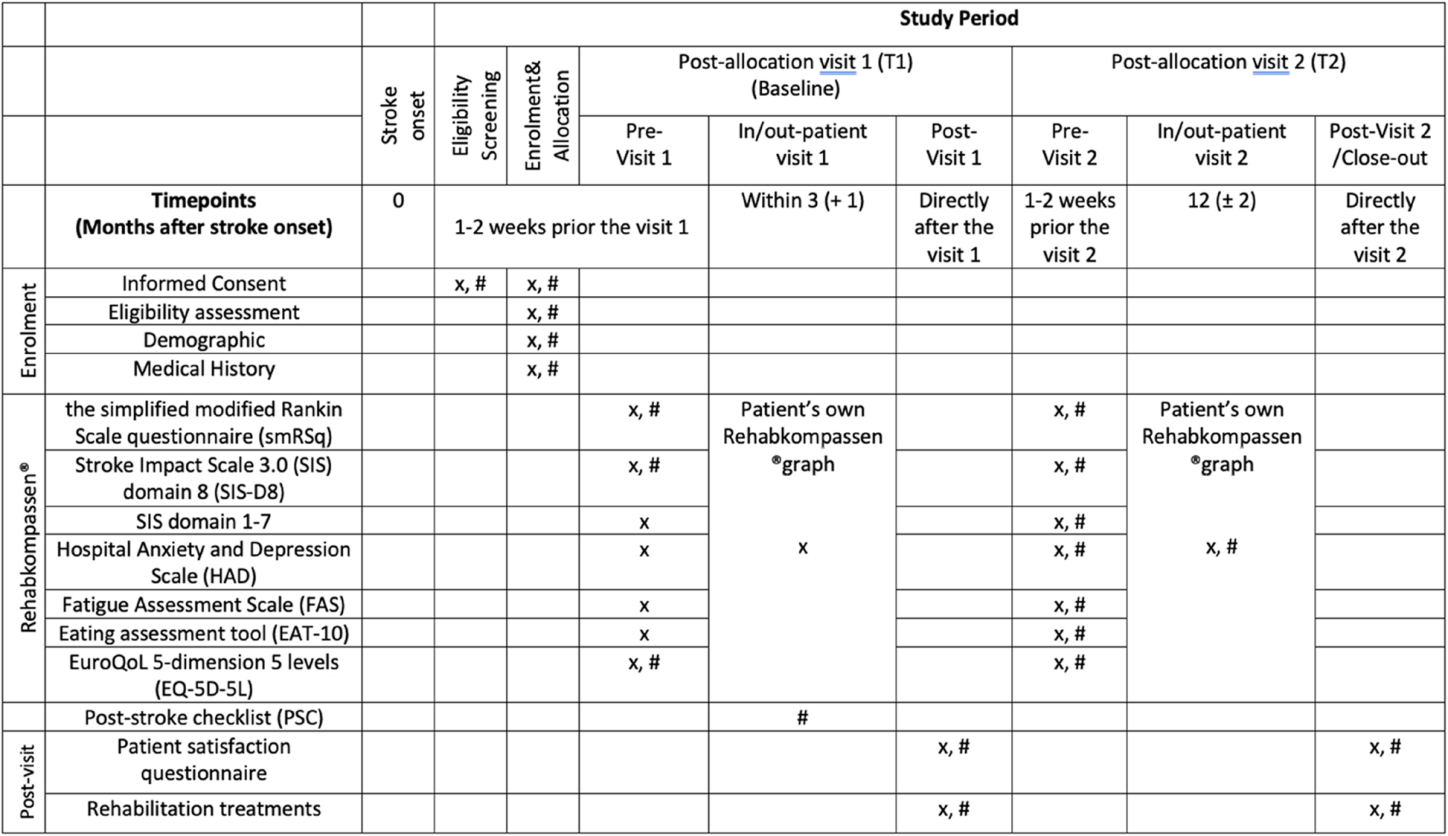
Schedule of enrolment, interventions, and assessments

x: for the patients in the intervention group; #: for the patients in the control group

#### Primary outcome

We will use two primary outcomes consisting of the smRSq [20–24] and Domain 8 of the SIS (SIS-D8) [25] since the smRSq covers daily activity and the SIS-Ds assesses social participation. This is because the smRSq, as a single primary outcome, is often not sensitive enough to capture the subtle alterations of treatment effects on stroke survivors [11, 21, 26]. Furthermore, our pilot study and previous Swedish stroke RCTs have demonstrated that most of the target study population has more limitations in social participation due to mild to moderate disability [11, 21, 27]. Domain 8 in the SIS (SIS-D8) [25] will thus be used as another primary outcome to capture the minor but important changes in social participation. Both primary outcomes will be reported by all patient-participants at the 3- and 12-month visits (Table 1). The time points for measurement of the primary outcomes will only be at 12 months after the occurrence of the stroke.

*Primary outcome: The smRSq* [20, 21] covers patients’ independence/disability levels in their daily activities. It is based on yes/no responses to five questions, which is summed into a seven-category total score (taking values from 0 to 6). An smRSq score of 0–2 (from no symptoms to independent but with a minor disability) will be considered a favourable outcome. A poor outcome will be considered as an smRSq score of 3–5 (from disability but able to walk to bed-bound and in need of full nursing care) or 6 (death). smRSq scores of 6 will be collected from patients’ medical records. The smRSq has shown good reliability among stroke survivors [20].

*Primary outcome: Domain 8 in the SIS (SIS-D8)* [25] assesses social participation (SIS-p), such as work and social activities. Social participation is the dominant problem among persons after stroke, as reported in previous Swedish stroke RCTs [21], but is not covered by the smRSq. The SIS-D8 will result in an ordinal score measuring social participation that ranges from 0 to 100. A higher score indicates a lower impact of stroke [28]. The SIS has demonstrated great internal consistency and construct validity in the stroke population [28, 29].

#### Secondary outcomes

Several secondary outcomes, including end-users’ satisfaction and feedback, barriers and facilitators for adopting the instrument, other stroke impacts, health-related quality of life and the cost-effectiveness of the instrument, will be assessed in the study (Table 1).

*Secondary outcomes: Patients’ satisfaction and feedback* will be assessed by the visit questionnaire consisting of 15 items concerning patients’ experiences using Rehabkompassen® [10, 11]. Each question will be answered on a Likert scale ranging from 1 to 5, with higher scores indicating better outcomes.

*Secondary outcomes: Clinicians’ experience and feedback* will be examined by:

1. The System Usability Scale [30], which consists of 10 items with five response options ranging from strongly agree to strongly disagree. The total score ranges from 0 to 100, with higher scores indicating better usability. The validity of the System Usability Scale has been demonstrated [31].
2. The Clinician Satisfaction Questionnaire, which consists of 17 items concerning clinicians’ experiences using Rehabkompassen® [10, 11]. Each question is answered on a Likert scale ranging from 1 to 5, with higher scores indicating better outcomes.
3. An implementation process evaluation according to the RE-AIM framework [32], which will be used to identify various factors at the individual and organizational levels that may be facilitators/barriers to further implementing Rehabkompassen®.

*Secondary outcomes: Stroke impacts* will be reported by the following PROMs:

- *Fatigue*: Fatigue will be measured by the FAS [33]. The FAS is a questionnaire used for identifying symptoms of chronic fatigue. It comprises 10 questions regarding both physical and mental fatigue that are answered on a scale from 1 (never) to 5 (always). The FAS has demonstrated convergent construct validity in stroke survivors [33, 34].
- *Dysphagia*: Dysphagia will be assessed by the EAT-10 [35], which includes 10 questions concerning swallowing difficulties. Each question is answered on a scale from 0 (no problems) to 4 (severe problems). The EAT-10 has shown structural validity among elderly individuals [36].
- *Depression and anxiety*: Depression and anxiety will be measured by the HADS. The HADS is a screening tool for the assessment of anxiety and depression. It comprises seven questions about anxiety and seven questions about depression that are answered on a scale from 0 (no symptoms) to 3 (severe symptoms). The subscales for anxiety and depression are added and interpreted separately. The HADS has excellent internal consistency and construct validity among stroke survivors [37].
- *Health status:* Health status will be assessed by the other 7 domains of the SIS [28], except for Domain 8, which was chosen as one of the primary outcomes. The SIS is a patient-reported, stroke-specific outcome measure containing 59 questions and a VAS for the estimation of perceived stroke recovery. As secondary outcomes, the proposed study will assess the impact of stroke within 7 domains, namely, strength, memory/cognition, feelings/emotions, communication, personal activities of daily living (ADL), instrumental ADL, mobility and motor impact. SIS data are presented as ordinal scores ranging from 0 to 100, and the higher the score is, the lower the impact of stroke [28].

*Secondary outcomes: Health-related quality of life* will be measured by the EQ-5D-5L [38]. The EQ-5D-5L consists of two parts: a visual analogue scale and a descriptive system covering five dimensions of health (mobility, hygiene, usual activities, pain/discomfort and anxiety/depression) with five response options (ranging from no problems to extreme problems). The latter can be translated to an index value with anchor points 1 (full health) and 0 (death) for eliciting the overall health utility score, corresponding to a quality-adjusted life year (QALY) score. Index values *less than 0* represent health states regarded as worse than death. The EQ-5D-5L has demonstrated construct and convergent validity among stroke survivors [39].

*Secondary outcomes: Cost-effectiveness* will be assessed in terms of cost per QALY, and resource utilization data, including the times and usage of medical resources around the patient visit, treatments or referrals initiated in conjunction with the visits, will be collected from medical journals by research staff.

Data on the end-users’ satisfaction and feedback will be collected from all patient-participants and medical staff at both visits, including participants’ and clinicians’ technical backgrounds, experiences and feedback regarding the use of Rehabkompassen® and resource utilization data. The time points for measurement of the end-users’ satisfaction and feedback will be at 3 and 12 months after the occurrence of the stroke. Other secondary outcomes will only be assessed in the intervention group at the 3-month visit and among all patient-participants at the 12-month visit, including various stroke impacts, including dysphagia, fatigue, depression and anxiety and motor or cognitive impacts (Table 1). Thus, the time points for measurement of other secondary outcomes will only be at 12 months after the occurrence of the stroke.

### Participant timeline {13}

The patient-participant timeline was created according to SPIRIT guidelines [13] and is presented in Table 1. After receiving written consent from the patient-participants, eligibility screening, enrolment and allocation will be carried out by the research staff at least 1 week before the first clinical visit (T1). During the first clinical visit, the participants will be allocated to either the intervention group (Rehabkompassen®) or the control group (the PSC) for the structured follow-up. Data collected at the first clinical visit will be used as baseline in the statistical analyses. The various rehabilitation interventions will subsequently be carried out according to their clinical routines in the respective clinics. At the second clinical visit (T2), all patient-participants will receive the intervention by using Rehabkompassen® during their follow-ups.

We enrolled our first participant on 7 February 2022. We expect to enrol the final participant at the end of 2024. The total recruitment period will be 3 years. Approximately 40% and 45% of the sample will be recruited in 2023 and 2024, respectively. The 12-month follow-up will be completed at the end of 2025, followed by data analysis, manuscript preparation and submission in 2026 (Table 2).

**Table 2:**
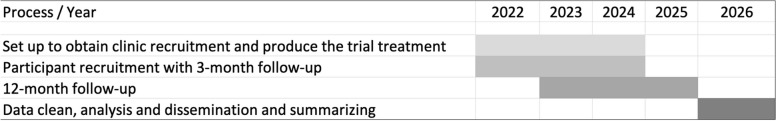
A GANTT schedule of the study

### Sample size {14}

The study is predicted to have 90% power in the analysis of both primary outcomes at a significance level of 2.5% for each individual test to account for planned correction for multiple comparisons with a target familywise error rate of 5%.

Sample size calculations were performed assuming a mean difference of 4 points in the SIS-p score between the groups at the 12-month follow-up. There is uncertainty regarding the minimum clinically important difference in the SIS-p, but differences in sizes between 10 and 15 points have previously been suggested [29]. A power analysis was conducted using Monte Carlo simulations, in which SIS-p data for the control group were generated from a beta distribution with *α* = 1.1 and *β* = 0.5, while Rehabkompassen® data were generated under the assumption of *α* = 1.1 and *β* = 0.385. This corresponds approximately to a mean value of 70 and a standard deviation of 28 for the control group, in line with what Guidetti et al. [25] reported for a population of stroke patients at 12 months after stroke. Under these assumptions, the results showed that we need to include 940 patients to detect a statistically significant difference in the SIS-p score when using an ordinal proportional odds model to compare the groups at the 12-month follow-up. The average odds ratio across all simulations was 1.5, as estimated using the function *orm* from the R package *rms* for comparing the intervention group to the control group [40]*.*

The sample size calculation for the mRS score was based on aggregated unpublished data from the Swedish Stroke Register (Riksstroke). The marginal distribution of mRS scores collected 12 months after stroke among Swedish patients was *0: 22.4%*, *1: 18.0%*, *2: 18.0%*, *3: 16.8%*, *4: 11.1%*, *5: 4.3%* and *6: 9.4%*. The function *popower* from the package *Hmisc* [41] in the statistical software R was used to determine that a sample size of 940 patients (470 in each group) is sufficient to detect a true odds ratio of 0.67 at 90% power when using an ordinal proportional odds model to compare groups.

Thus, 940 patients should be sufficient for both primary outcomes. To account for a 15% loss to follow-up rate, we plan to recruit 1106 patients for the study (Fig. 3).

### Recruitment {15}

To achieve the target sample size, patients will be recruited from approximately 15 participating outpatient clinics throughout Sweden. The participating clinics have been and will be recruited either by personal contacts or after the study is promoted at the national and regional conferences.

To attract more clinics to participate in the study, the Chief Investigator will continuously promote the study at professional and lay-community conference/educational events. To attract more patients, recruitment posters with the contact details of research staff will be displayed at each participating site. All stroke survivors in the participating clinics will receive the study information and a letter inviting them to participate in the study. Other study awareness efforts, including clinical trial finder websites and word of mouth, will also be used to enhance the recruitment of clinics and participants. Moreover, recruitment status relative to planned targets will be reviewed monthly by the administrative study team, and the Chief Investigator will adjust the recruitment plans as needed.

## Assignment of interventions: allocation

### Sequence generation {16a}

The allocation sequence will be created centrally using computed-generated random numbers stratified by clinic. To reduce predictability, permuted block randomization will be used with random block sizes from 2 to 8 to ensure that participants are randomly assigned to the intervention or control group [42].

### Concealment mechanism {16b}

A statistician (the second author) who is not involved in outcome assessment or the patients’ treatment will generate the allocation sequence. The allocation sequence will be installed in REDCap, which will be concealed from the study personnel working in participant recruitment. The study personnel in each clinic will automatically receive an allocation decision with a study number when they register a new patient in REDCap.

### Implementation {16c}

Research staff at the local clinic will enrol participants via REDCap, which will automatically assign participants to either the control or intervention group.

## Assignment of interventions: blinding

### Who will be blinded {17a}

As in many rehabilitation intervention studies, this is an open-label study in which a double-blind intervention is not possible. Group allocation will be blinded in data analysis by two statisticians.

### Procedure for unblinding if needed {17b}

The design is open label with only data analysts being blinded so unblinding will not occur.

## Data collection and management

### Plans for assessment and collection of outcomes {18a}

We will use 6 validated and reliable PROMs as the outcome measurements in the study (see Table 1 and the “Outcomes {12}” section) to facilitate the development of patient-tailored rehabilitation regimens, strengthen patient-centred care and stitch together the currently fragmented post-acute continuum of care after stroke. To promote data quality, all clinical staff who participate in the study will be authorized, trained and competent according to the ethically approved protocol, the principle of Good Clinical Practice (GCP). The Chief Investigator and two research nurses will provide support whenever needed by clinical staff.

### Plans to promote participant retention and complete follow-up {18b}

We will employ several strategies to promote complete participant follow-up. A free clinical visit at approximately 12 months after stroke onset is an important promotor to enhance participant retention. Before the 3- and 12-month visits, an appointment date will be sent to the participants via regular mail, which will be followed by a phone call from research personnel. Moreover, all participants will receive the Rehabkompassen**®** questionnaires in their inbox at 1177.se. Directly after the 12-month visit, a feedback questionnaire will be provided digitally via 1177.se. If the response is not provided within a week, research personnel will make a phone call to remind the participants. In the case of no response, research personnel may call the participant again. For participants who experience technical difficulties, technical assistance will be provided by research personnel when needed. In addition, the option of video visits is also available in case a participant is not able to have a face-to-face visit.

### Data management {19}

Rehabkompassen will collect PROM data automatically, which will be kept securely in electronic form in the Region of Västerbotten with regular backups. Baseline data collection will be carried out by using REDCap, hosted at Umeå University. REDCap [19] is a secure, web-based software platform designed to support data capture for research studies. It provides several functions, such as (1) an intuitive interface for validated data capture; (2) audit trails for tracking data manipulation and export procedures; (3) automated export procedures for seamless data downloads to common statistical software packages; and (4) procedures for data integration and interoperability with external sources [19]. All data will be coded with a study number, the participant’s initials and the participant’s date of birth. Paper coding files will be stored in a secure location at each site. A separate data management plan for the study is kept at Umeå University.

### Confidentiality {27}

All study personnel will endeavour to protect the rights of the participants to privacy and informed consent. All information collected will be treated confidentially in accordance with the consent provided, adhering to the EU data protection rules (https://ec.europa.eu/info/law/law-topic/data-protection/eu-data-protection-rules_en). No outsider will be able to access the database. All analyses will take place at the group level where no individuals can be identified. Upon study completion, the data will be transferred from Redcap to Umeå University in an encrypted format and stored for at least 10 years together with data from Rehabkompassen®.

### Plans for collection, laboratory evaluation and storage of biological specimens for genetic or molecular analysis in this trial/future use {33}

This is not applicable as no biological specimens will be collected.

## Statistical methods

### Statistical methods for primary and secondary outcomes {20a}

Group differences in the mRS and SIS-p scores at the 12-month follow-up will be tested using ordinal logistic (proportional odds) regression, adjusted for site with fixed effects [43]. For the SIS-p score, models will also be adjusted for the baseline SIS-p score, with natural cubic splines to account for nonlinearity in the association. The results from the analyses will be presented as odds ratios with corresponding 95% confidence intervals and *p* values.

For the two primary outcomes, adjustments of the significance levels of the individual tests will be performed using the Holm–Bonferroni method to ensure that the familywise error rate will not be inflated above 0.05.

Secondary outcomes on an ordinal scale will be analysed similarly using ordinal logistic regression. All analyses will primarily be conducted in accordance with the intention-to-treat principle. As complementary analyses, per-protocol analyses will be performed using data from patients with full availability of primary variables and predefined criteria for adherence. Further details about the statistical analyses will be predefined and published in a separate statistical analysis plan. A data management plan has been established, providing details of the handling, organization and storage of study data.

A statistical significance level of *p* < 0.05 (two-tailed) will be applied to all analyses. For the two primary outcomes, adjustments of the significance levels of the individual tests will be performed using the Holm–Bonferroni method to ensure that the familywise error rate will not be inflated above 0.05.

### Interim analyses {21b}

No interim analyses are planned.

### Methods for additional analyses (e.g. subgroup analyses) {20b}

Subgroup analyses, e.g. based on age (< 65 years, 65–80 years and > 80 years), sex (male and female), stroke severity (NIHSS score; mild, 0–8 moderate, 9–15 and severe, > 15), stroke type (ischaemic vs. haemorrhagic), treatment with tissue plasminogen activator and/or thrombectomy (yes or no) and geographical region, will be conducted as complementary analyses.

### Methods in analysis to handle protocol nonadherence and any statistical methods to handle missing data {20c}

All analyses will be conducted in accordance with the intention-to-treat principle. Multiple imputation of missing data will be employed in the primary analysis using multiple imputation by chained equation (MICE) [44]. As sensitivity analyses, per-protocol analyses will be performed using data from patients with fully available data for the primary variables and no protocol violations. The per-protocol dataset will be predefined in the statistical analysis plan of the study.

### Plans to give access to the full protocol, participant-level data and statistical code {31c}

The study protocol, including statistical analyses, will be available in conjunction with the scientific publication. Upon study completion, anonymized data will also be available to the scientific community at large through publications. Moreover, any party may apply to the Chief Investigator for access to the full protocol, deidentified participant-level data and the statistical code for academic research purposes by a study collaboration request. The steering committee will govern data access.

## Oversight and monitoring

### Composition of the coordinating centre and trial steering committee {5d}

The University Hospital of Umeå is the coordinating centre where the Chief Investigator (the first author) and a trial management group (TMG) work. The coordinating centre oversees the fiscal management of the study, ethics approvals, subcontracts with other participating clinics and other financial and oversight responsibilities. The TMG consists of two research nurses, two medical engineers, two RCT coordinators and the Chief Investigator. The TMG provides daily support and troubleshooting to the local participating clinics and has a brief meeting once a week to discuss actual issues.

A trial steering committee (TSC) is composed of a national collaboration among four universities and regions in Sweden. The TSC includes an independent chairperson (the last author) and multiple independent members (coauthors), including statisticians, persons with biomedical engineering degrees, rehabilitation and trial experience and patient representatives (nonauthors). TSC responsibilities include clinical setup of the study, ongoing management, study promotion and planning for the interpretation and dissemination of results. TSC meetings were held monthly during the trial planning phase. TSC meetings will take place annually at a minimum to monitor study progress and provide public, clinical information during the active phase. Moreover, professional advice to TMG members will be provided in a timely manner via e-mail or telephone when needed. The TSC will be responsible for reviewing issues and any concerns warranting modification or termination of the study.

An advisory board consisting of three professors within the stroke field will provide supervision and feedback on the project annually to the TSC. Together with TSC, this board will ensure that the interpretation of findings is applicable to current practice.

### Composition of the data monitoring committee, its role and reporting structure {21a}

The data monitoring committee (DMC) comprises two RCT process controllers who are independent of the study sponsor and funder. The DMC will monitor clinical trial compliance, the completeness of the study data and data safety twice a year, in accordance with the Standard Operating Procedure (SOPs) and Consolidated Standards of Reporting Trials (CONSORT) statement.

### Adverse event reporting and harms {22}

Even though they are unlikely in this study, adverse events will be immediately reported to the TSC if classified as Related and Unsuspected Serious Adverse Events (RUSAEs), i.e. events that are unexpected in severity and seriousness and suspected to be related to the study intervention. The TSC will then report the case to the DMC with a tailored action plan.

### Frequency and plans for auditing trial conduct {23}

To increase the fidelity of the study, the DMC members who are independent of the sponsor will regularly (4 times while participating in the study) notify the participating clinics if there is a breach of protocol or GCP principles that is likely to significantly affect participant safety, health and wellbeing as well as the scientific value of the research.

### Plans for communicating important protocol amendments to relevant parties (e.g. trial participants, ethical committees) {25}

Important protocol amendments will be made by the Chief Investigator following consultation with the TMG and the TSC. All changes to the protocol will be reviewed and approved by the Swedish Ethical Review Authority prior to their implementation. Amendments will then be communicated to research staff and participants by the PI. The major protocol changes will be updated by the Chef Investigator in the clinicaltrials.gov and ISRCTN registry protocol registration within 14 days.

## Dissemination plans {31a}

Annual trial meetings will be organized for all unit-allied health professionals for exchanging experiences and spreading knowledge. Monthly newsletters and ongoing e-mail contact will also be used to update the study progress and facilitate communication during the study.

Upon completion of the trial, investigators will report results, regardless of the direction or magnitude of the effect, through peer-reviewed journal articles, scientific presentations, patient education websites, public media, ClinicalTrials.gov and ISRCTN registry. We endeavour to facilitate knowledge transfer and exchange among different stakeholders.

## Discussion

Stroke is a leading cause of disability among adults worldwide, with a heavy burden for patients and their families as well as society [2, 3]. Despite the recent recommendation by Swedish stroke guidelines, structured follow-up to identify patients’ rehabilitation needs and develop patient-tailored rehabilitation regimens is largely lacking in current stroke care [4]. Establishing such care, however, risks heavy encumbrances for our already time- and resource-constrained health care system. Therefore, a cost-effective and user-friendly solution is desperately needed to improve patients’ daily activities and quality of life.

To meet these challenges, Rehabkompassen® was recently created and developed as a novel and unique digital follow-up tool to identify and graphically visualize a panoramic view of stroke patients’ heterogeneous rehabilitation needs based on 6 well-validated and reliable PROMs [10, 11]. The utilization of Rehabkompassen® may facilitate the identification of rehabilitation needs among persons after stroke and therefore advance patient-tailored rehabilitation regimens and improve patients’ clinical outcomes. In addition to the intervention arm, an active control arm is planned by using the paper form of the PSC [17] as a structured follow-up tool, recommended by the Swedish National Board of Health and Welfare during clinical visits in usual care settings. This will ensure that the patients in the control group will receive equal care. Since the PSC has not been widely used in usual stroke care in Sweden, this trial could promote the usage of the PSC.

This two-arm pragmatic multisite study will be able to provide unequivocal results of the eventual effectiveness of the Rehabkompassen tool since both the intervention and control are used in normal practice. Moreover, this trial provides substantial flexibility concerning the adaptation of the Rehabkompassen tool into everyday clinical practice, such as stroke units, early support discharge units and stroke rehabilitation units, in the post-acute continuum of care after stroke in Sweden. A multicentre study also means extra attention should be given to ensure the minimization of the differences and variations among clinics. This is why we will collect baseline data concerning usual care and provide the study information and a training program on the usage of Rehabkompassen® and REDCap prior to patient recruitment. In addition, much information about implementation will be collected from medical practitioners in the participating clinics in the proposed study. Together with the high generalizability of the pragmatic trial [14], this will facilitate the future implementation of the Rehabkompassen® tool in combination with usual care as a standard structured follow-up model in the rest of the country if successful.

Despite the potential usefulness of the tool, there are various practical and operational issues to consider in this study. We are certainly aware of the major challenge of recruiting more than 1000 patients, which is required for the power analysis. Our pilot study demonstrated a recruitment rate of 28% with a retention rate of 86% at the 12-month follow-up without any severe adverse effects during the heavy pandemic period [11]. This means that we need to screen approximately 5000 stroke patients, i.e. approximately 8% of the total stroke patients in Sweden, during 3 years of recruitment. This is why we are confident that recruiting 1106 patients is still an achievable goal, especially with strong financial support from various Swedish research funding.

We are also aware of the challenge of running such a large multicentre study, especially concerning data collection and management, which is why a separate data management plan has been established and kept at Umeå University. Another issue is technical obstacles for the participants, who are often elderly since the mean age of stroke patients is 73 years old [11]. We have thus planned to have research nurses or a nurse assistant who will assist participants in overcoming technical issues in each county. Finally, the challenge of unblinding the intervention will increase the risk of spill-over effects when patients from the treatment and control groups are treated in the same clinic. GCPs, including strictly keeping the research protocol separate for the intervention and control groups, will decrease the risk of possible spill-over effects.

This study will provide strong evidence on the effectiveness and cost-effectiveness of a novel digital follow-up tool, Rehabkompassen®, in persons discharged from acute care settings after stroke. In addition to improving the tool to be more user -riendly, we envisage finding a cost-effective solution to carry out standard follow-up visits and to facilitate patient-tailored rehabilitation in persons with stroke in current clinical practice. This tool has the potential to improve everyday life for many stroke patients and to reduce the health, social and economic burdens after stroke for patients and their families as well as society. The outcomes of this trial will inform clinical practice and health care policy on the role of the digital follow-up tool Rehabkompassen® in the post-acute continuum of care after stroke.

## Trial status

The Rehabkompassen® trial is currently being conducted under version 5.0 of the protocol (2023–06-26. We enrolled our first participant on 2022–02-07. The final participant is scheduled to be enrolled in December 2025 and will complete the study by the end of 2026.

## Data Availability

Key study investigators will have access to the final trial dataset. Trial data will be made available to nonstudy investigators following the approval of a study collaboration request by TSC.

## Abbreviations

DMC: Data monitoring committee
EAT-10: Eating Assessment Tool
EQ-5D-5L: EuroQoL 5-dimension 5-level
FAS: Fatigue Assessment Scale
GCP: Good Clinical Practice
HAD: Hospital Anxiety and Depression Scale
mRS: Modified Rankin Scale
PROMs: Patient-reported outcome measures
ePROMs: Electronic PROMs
PSC: Post-Stroke Checklist
RCT: Randomized controlled trial
SIS: Stroke Impact Scale
SIS-p: SIS score of social participation
smRSq: Simplified modified Rankin Scale questionnaire
TSC: Trial steering committee
TMG: Trial management group
